# *Staphylococcus aureus* From Goats Are Genetically Heterogeneous and Distinct to Bovine Ones

**DOI:** 10.3389/fvets.2020.00628

**Published:** 2020-09-09

**Authors:** Alicia Romanò, Alessandra Gazzola, Valentina Bianchini, Claudia Cortimiglia, Antonio M. Maisano, Paola Cremonesi, Hans U. Graber, Fausto Vezzoli, Mario Luini

**Affiliations:** ^1^Istituto Zooprofilattico Sperimentale della Lombardia e Dell'Emilia-Romagna, Lodi, Italy; ^2^Agroscope, Research Division, Food Microbial Systems, Bern, Switzerland; ^3^Department of Veterinary Medicine, Università degli Studi di Milano, Milan, Italy; ^4^Institute of Agricultural Biology and Biotechnology, National Research Council, Lodi, Italy

**Keywords:** *Staphylococcus aureus*, mastitis, goat, small ruminants, genotyping, virulence gene profile, Italy

## Abstract

*Staphylococcus aureus* is one of the major pathogens responsible for intramammary infections in small ruminants, causing severe economic losses in dairy farms. In addition, *S. aureus* can contaminate milk and dairy products and produce staphylococcal enterotoxins, being responsible for staphylococcal food poisoning. Currently, data on the population structure and the virulence gene patterns of *S. aureus* strains isolated from goat milk is limited. Therefore, this study aimed at defining Ribosomal Spacer PCR (RS-PCR) genotypes, clonal complexes (CC), *spa* types, and virulence gene profiles of *S. aureus* isolated from goat milk samples from Lombardy region of Italy. A total of 295 *S. aureus* isolates from 65 goat bulk tank milk samples were genotyped by RS-PCR. *spa* typing and virulence gene patterns of a subgroup of 88 isolates were determined, and MLST was performed on a further subgroup of 39 isolates, representing all the *spa* types identified during the analysis. This study revealed 7 major genotypic clusters (CLR, CLAA, CLZ, CLAW, CLBW, CLS, and CLI), of which *S. aureus* CLR (19.8%) was the most common. A total of 26 different *spa* types were detected, the most prevalent types were t1773 (24%), t5428 (22.7%), and t2678 (12.5%). Overall, 44.3% of all isolates harbored at least one enterotoxin gene. The most prevalent was the combination of *sec*-*sel* genes (35.2%). Based on their MLST, isolates were assigned to 14 different CC, with majority grouped as CC133 (24%), CC130 (19.6%), and CC522 (19.6%). The caprine *S. aureus* population was depicted with a minimum spanning tree and an evolutionary analysis based on spa typing and MLST, respectively. Then, the variability of such strains was compared to that of bovine strains isolated in the same space-time span. Our results confirmed that *S. aureus* isolates from goats have wide genetic variability and differ from the bovine strains, supporting the idea that *S. aureus* from small ruminants may constitute a distinct population.

## Introduction

*Staphylococcus aureus* is one of the major pathogens responsible for clinical and subclinical mastitis in goats, causing significant economic losses due to the decrease of quality and quantity of milk and to the treatment losses for staphylococcal mastitis. Furthermore, *S. aureus* can contaminate both raw milk and dairy products, being a source of food poisoning (1, 2).

The prevalence of *S. aureus* in dairy goat herds, estimated by analyzing bulk tank milk, varies between 16.7 and 96.2% in different countries (3–5). In Italy, the prevalence of *S. aureus* in dairy goat herds was reported to be 43.1% in the Lombardy Region (6) and 76.9% in Sardinia (7).

Ribosomal Spacer PCR (RS-PCR) is an accurate, rapid, inexpensive genotyping method with high discriminatory power (8). It has been used to classify *S. aureus* isolates into genotypes, that are inferred from the electrophoresis profile using the Mahal software, according to Fournier et al. (9) and Graber (10). In particular, an electrophoretic profile differing in more than one band from all identified genotypes is considered as a new genotype. Genotypes are named and extended leading to the genotypes GTA to GTZ, followed by the genotypes GTAA to GTAZ, GTBA to GTBZ, and so on. Genotypic variants, which differ in only one band from the genotype after the electrophoretic analysis, were indicated with roman superscripts (e.g., GTR^I^) (10). RS-PCR genotypes, in turn, can be grouped into clusters (CL), each of which consists of a genotype and all its variants (e.g., GTR and GTR^I^, GTR^II^, GTR^III^, etc.).

Several studies performed RS-PCR to genotype *S. aureus* strains isolated from bovine milk, and showed a great genotypic variety, differing in their contagiousness (8, 9, 11, 12). *S. aureus* CLB was found to be highly contagious and has been associated with high within-herd prevalence of intramammary infections (IMI), together with CLR and CLS (8, 9, 12). In contrast, *S. aureus* CLC appears to be less problematic because it generally affects individual cows and only one quarter of the mammary gland is affected (11, 13). Moreover, each genotype presented a different pattern of virulence genes (9, 14, 15). In spite of this detailed knowledge on dairy-associated *S. aureus* genotypes, no data on *S. aureus* genotypes isolated from goats is currently available.

Previous studies suggest that the lineages CC130, CC133, and CC522 represent the major CC among *S. aureus* isolates from milk of small ruminants (4, 15–17). *S. aureus* isolates from caprine and ovine hosts have been *spa* typed previously (15–18). In goats and sheep, fewer *spa* types were found compared to cows (15). Merz et al. (17) reported t1773 (corresponding to CC130) as the most prevalent *spa* type in caprine isolates.

*S. aureus* produces a wide range of virulence factors, which are essential for a successful infection. At least 25 different toxins (e.g., enterotoxins SEA to SEQ, toxic shock syndrome toxin-1 *tst1*, exfoliative toxins ETA and ETB), 15 microbial surface components recognizing adhesive matrix molecules (such as clumping factor A *clfA*), 20 immune evasion molecules (such as protein A, coagulase, hemolysins, and leukocidins), among others are known *S. aureus* virulence factors (19). The most prevalent enterotoxin genes detected in *S. aureus* isolated from small ruminants were *sec* and *sel*, whereas the *sea* gene was found exclusively in caprine isolates (15, 17). Merz et al. (17) reported that all the isolates harboring the *tst1* gene also harbored the combination of genes *sec*-*sel* and were assigned exclusively to CC130 and CC133. Significantly higher prevalence rates of *sec, sel*, and *tst1* genes were observed in *S. aureus* from small ruminants than among bovine isolates (15, 17).

While the population structure and the genomic characteristics of *S. aureus* isolated from bovine milk are very well-described, similar data on caprine *S. aureus* is very limited. Therefore, this study aimed at defining RS-PCR genotypes, clonal complexes (CC), *spa* types, and virulence gene profiles of *S. aureus* isolated from goat milk in the Lombardy region in northern Italy.

## Materials and Methods

### Bacterial Strains and DNA Extraction

*S. aureus* isolated from goat bulk tank milk (BTM) collected by Cortimiglia et al. (6) was used for this study, thus the sampling criteria and bacteriological analysis have already been described. Briefly, BTM samples were collected from 197 different dairy goat farms located in Lombardy (northern Italy) between July and October 2012. After collection, milk samples were transported at +4°C to the laboratory and stored at −20°C till analysis, which were conducted within 3 months. Bacteriological analysis was performed with standard techniques for *S. aureus* isolation, as previously described by Cortimiglia et al. (6). In particular, milk samples were spread on blood agar supplemented with 5% defibrinated sheep blood and on Baird Parker agar supplemented with rabbit plasma fibrinogen (BP-RPF; Oxoid Ltd., Basingstoke, UK), plates were incubated at 37°C and analyzed after 24 and 48 h. Colonies developing a typical coagulase halo on BP-RPF agar were considered suspected *S. aureus*, as were hemolytic colonies on BA testing positive in a tube coagulase test (Coagulase plasma-EDTA, Biolife srl, Milan, Italy).

DNA was extracted using the DNA isolation system kit (Clonit, Medical System, Genova, Italy) according to the manufacturer's guidelines. DNA quality and quantity were measured using a NanoDrop ND-1000 spectrophotometer (Nano-Drop Technologies, Wilmington, DE), and stored at −20°C till further use.

### RS-PCR

If present, five *S. aureus* isolates from each BTM positive sample were genotyped by RS-PCR, according to Fournier et al. (9) and Graber (10). When different cultural morphologies were detected (pigmentation and hemolysis on blood agar or coagulase and lecitinase halos on Baird Parker agar), four colonies per morphology were selected in each sample.

The PCR products were analyzed using the miniaturized electrophoresis system DNA 7500 LabChip [Agilent Technologies, Santa Clara, CA; (9, 10)]. Genotypes were inferred from the electrophoresis profile using the Mahal software, which is freely available online (10)^1^.

### *Spa* Typing

Typing of *spa* was performed on a subset of strains, representing all the *S. aureus* RS-PCR genotypes found in each milk sample (i.e., herd). In detail, one or more isolates were selected from each BTM sample, depending on the number of different genotypes identified with the RS-PCR. Typing was performed according to Shopsin et al. (20), and *spa* types were assigned using the code system described on Ridom SpaServer^2^.

To depict the frequency and genetic relatedness among the isolates, the *spa* typing results were analyzed, and a minimum spanning tree (MST) was constructed using the BioNumerics 7.6 software (Applied Maths, Sint-Martens-Latem, Belgium).

### Virulence Factors

PCR amplifications were performed on the same strains selected for *spa typing* to investigate the presence of 22 virulence factors contributing to *S. aureus* pathogenicity: enterotoxins (from *sea* to *see*, and from *seg* to *sel*) (21, 22), toxic shock syndrome (*tst*) and exfoliative (*eta, etb*) toxins (21), PVL (*lukS-lukF*) (23), leucocidin M (*lukM*), and leukotoxin ED (*lukE-lukD*) (24), cell-wall associated protein *clfA* (clumping factor A) (21), *cna* (collagen-binding protein) (25), and *fmtB* (cell-wall protein), *scn, chp*, and *sak* belonging to the immune evasion cluster (IEC) (26). The primers and protocols are listed in the Supplementary Table.

### MLST

Multi Locus Sequence Typing (MLST) was performed as previously described (27) on one representative strain for each combination of *spa* typing and RS-PCR genotyping result. Allele numbers and sequence type (ST) were assigned as per the MLST database^3^ Clonal analysis of the STs was performed using the clustering algorithm e-BURST^4^

An evolutionary analysis was carried out using goeBURST^5^ (Algorithm 3.0), a java implementation of the eBURST algorithm rules proposed by Feil et al. (28), using a graphic matroid approach that ensures an optimal solution for the placement of links between STs.

### Statistical Analysis

The degree of similarity between the distributions of RS-PCR genotypes among caprine strains and bovine *S. aureus* strains in the same geographical area and during the same time period (12) was estimated using the Czekanowski index or Proportional Similarity Index (PSI). It is calculated by

(1) $$\begin{array}{l} PSI=1-0.5\sum_{i}^{\;}\left|p_{i}-q_{i}\right|\;=\;\sum_{i}^{\;}\min\;(p_{i},q_{i}) \end{array}$$

in which *p*_*i*_ and *q*_*i*_ represent the proportion of genotypes in the two distinct populations (caprine and bovine *S. aureus* strains), while *i* represents the total numerosity of the two populations. The values for PSI range from 1 for identical frequency distributions of the variable of interest to 0 for no similarities between the data sets (29).

## Results

### RS-PCR

A total of 295 *S. aureus* isolates from 65 out of 197 examined goat BTM samples were genotyped by RS-PCR. The resulting genotypes with their variants were grouped into clusters, obtaining 29 genotypes grouped into 18 clusters (Table 1). A single genotype was detected in 43 samples (66.2%), whereas 2 and 3 different genotypes were identified in 18 (27.7%) and 4 (6.2%) samples, respectively. The analyzed isolates were grouped into 7 major CL, in the following order of prevalence: CLR (18/65; 27.7%), CLAA (16/65; 24.6%), CLZ (13/65; 20%); CLAW (11/65; 16.9%); CLBW (9/65; 13.8%), CLS (7/65; 10.8%), and CLI (2/65; 3.1%). Each one of the remaining 11 minor CL was isolated in only one sample.

**Table 1:** RS-PCR genotypes and genotypic clusters of *Staphylococcus aureus* isolated from goat bulk tank milk samples, sorted by frequency.

**Cluster**	**Number of isolates**	**Genotype**
CLR	18	R^XIII^ (7), R^II^ (4), R (3), R^VII^ (3) R^I^ (1)
CLAA	16	AA (16)
CLZ	13	Z (8), Z^II^ (5)
CLAW	11	AW^I^ (6), AW (3), AW^II^ (2)
CLBW	9	BW^II^ (6), BW (2), BW^I^ (1)
CLS	7	S (6), S^I^ (1)
CLI	2	I^I^ (1), I^II^ (1)
CLAL	1	AL
CLAX	1	AX
CLB	1	B
CLBE	1	BE
CLBJ	1	BJ
CLBS	1	BS^III^
CLBX	1	BX
CLBY	1	BY
CLBZ	1	BZ
CLO	1	O
CLW	1	W
CLND	1	ND

Thirty genotypes were found, belonging to 19 clusters.

*ND, not determined*.

### *Spa* Typing

Typing of *spa* was performed on a subset of 88 strains. A total of 26 different *spa* types were detected and the most prevalent types were t1773 (*n* = 21; 23.9%), t5428 (*n* = 20; 22.7%), and t2678 (*n* = 11; 12.5%). Other *spa* types, such as t7630, t899 (*n* = 4; 4.5%), t1180, t988 (*n* = 3; 3.4%), and t524, t13986 (*n* = 2; 2.3%), were less frequent, and each of the remaining 17 *spa* types was detected only once (Table 2).

**Table 2:** Molecular characteristics of the 88 *Staphylococcus aureus* strains isolated from goat bulk tank milk.

	**Genotyping**			**Virulence profile**												
**N**.	**RS-PCR Cluster**	***spa* type**	**MLST**	**Enterotoxins**	***tst***	***eta***	***etb***	***scn***	***sak***	***chp***	***fmtb***	***cna***	***clfA***	***lukE-lukD***	***lukM***	***pvl***
1	CLR	t1773	CC130-ST3044	*sed, sej*	–	–	–	–	–	–	+	+	+	+	+	–
2	CLR	t1773	nd	*sed, sej*	–	–	–	–	–	–	+	+	+	+	–	–
3	CLR	t1773	nd	*sec, sel*	+	–	–	–	–	–	+	+	+	+	+	–
4	CLR	t1773	nd	*sec, sel*	+	–	–	–	–	–	+	+	+	+	+	–
5	CLR	t1773	nd	*sec, sel*	+	–	–	–	–	–	+	+	+	+	+	–
6	CLR	t1773	nd	*sec, sel*	+	–	–	–	–	–	+	–	+	+	+	–
7	CLR	t1773	nd	–	–	–	–	–	–	–	+	+	+	+	–	–
8	CLR	t1773	nd	–	–	–	–	–	–	–	+	–	+	+	–	–
9	CLR	t1773	nd	–	–	–	–	–	–	–	+	–	+	+	+	–
10	CLR	t2678	CC133-ST133	*sec, sel*	+	–	–	–	–	–	+	+	+	+	+	–
11	CLR	t2678	nd	*sec, sel*	+	–	–	–	–	–	+	+	+	+	+	–
12	CLR	t2678	nd	*sec, sel*	–	–	–	–	–	–	+	–	+	+	+	–
13	CLR	t203	CC352-ST352	–	–	–	–	–	–	–	+	+	+	+	+	–
14	CLR	t1180	CC133-ST3046	*sec, sel*	+	–	–	–	–	–	+	+	+	+	+	–
15	CLR	t6106	CC133-ST133	*sec, sel*	+	–	–	–	–	–	+	+	+	+	+	–
16	CLR	t12032	CC133-ST133	*sec, sel*	+	–	–	–	–	–	+	–	+	+	+	–
17	CLR	t13987	CC130-ST3083	*sec, sel*	+	–	–	–	–	–	+	+	+	+	+	–
18	CLR	unknown	Singleton-ST3082	*sec, sel*	+	–	–	–	–	–	+	–	+	+	+	–
19	CLAA	t5428	CC522-ST522	–	–	–	–	–	–	–	+	+	+	+	–	–
20	CLAA	t5428	nd	–	–	–	–	–	–	–	+	+	+	+	–	–
21	CLAA	t5428	nd	–	–	–	–	–	–	–	+	+	+	+	–	–
22	CLAA	t5428	nd	–	–	–	–	–	–	–	+	+	+	+	–	–
23	CLAA	t5428	nd	–	–	–	–	–	–	–	+	+	+	+	–	–
24	CLAA	t5428	nd	–	–	–	–	–	–	–	+	+	+	+	–	–
25	CLAA	t5428	nd	–	–	–	–	–	–	–	+	+	+	+	–	–
26	CLAA	t5428	nd	–	–	–	–	–	–	–	+	+	+	+	+	–
27	CLAA	t5428	nd	–	–	–	–	–	–	–	+	+	+	+	+	–
28	CLAA	t5428	nd	–	–	–	–	–	–	–	+	+	+	+	+	–
29	CLAA	t5428	nd	–	–	–	–	–	–	–	-	+	+	+	+	–
30	CLAA	t5428	nd	–	–	–	–	–	–	–	+	+	+	+	+	–
31	CLAA	t7630	CC522-ST3169	–	–	–	–	–	–	–	+	+	+	+	+	–
32	CLAA	t7630	nd	–	–	–	–	–	–	–	+	+	+	+	-	–
33	CLAA	t13986	CC522-ST522	–	–	–	–	–	–	–	+	+	+	+	+	–
34	CLAA	t13986	nd	–	–	–	–	–	–	–	+	+	+	+	–	–
35	CLZ	t2678	CC133-ST701	*sec, sel*	+	–	–	–	–	–	+	+	+	+	+	–
36	CLZ	t2678	nd	*sec, sel*	+	–	–	–	–	–	+	+	+	+	+	–
37	CLZ	t2678	nd	*sec, sel*	+	–	–	–	–	–	+	+	+	+	+	–
38	CLZ	t2678	nd	*sec, sel*	+	–	–	–	–	–	+	+	+	+	+	–
39	CLZ	t2678	nd	*sec, sel*	+	–	–	–	–	–	+	–	+	+	+	–
40	CLZ	t2678	nd	*sec, sel*	+	–	–	–	–	–	+	–	+	+	+	–
41	CLZ	t2678	nd	*sec, sel*	+	–	–	–	–	–	+	–	+	+	+	–
42	CLZ	t988	CC133-ST133	*sec, sel*	+	–	–	–	–	–	+	+	+	+	+	–
43	CLZ	t998	nd	*sec, sel*	–	–	–	–	–	–	+	+	+	+	+	–
44	CLZ	t1180	CC133-ST3046	*sec, sel*	+	–	–	–	–	–	+	+	+	+	+	–
45	CLZ	t1180	nd	*sec, sel*	+	–	–	–	–	–	+	–	+	+	+	–
46	CLZ	t1773	CC130-ST3044	–	–	–	–	–	–	–	+	–	+	+	+	–
47	CLZ	t14053	CC133-ST133	*sec, sel*	+	–	–	–	–	–	+	–	+	+	+	–
48	CLAW	t1773	CC130-ST3044	*sec, sel*	+	–	–	–	–	–	+	+	+	+	+	–
49	CLAW	t1773	nd	*sec, sel*	+	–	–	–	–	–	+	+	+	+	–	–
50	CLAW	t1773	nd	*sec, sel*	+	–	–	–	–	–	+	+	+	+	–	–
51	CLAW	t1773	nd	–	–	–	–	–	–	–	+	+	+	+	–	–
52	CLAW	t1773	nd	–	–	–	–	–	–	–	+	+	+	+	–	–
53	CLAW	t1773	nd	–	–	–	–	–	–	–	+	+	+	+	–	–
54	CLAW	t1773	nd	–	–	–	–	–	–	–	+	+	+	+	+	–
55	CLAW	t1773	nd	–	–	–	–	–	–	–	+	–	+	+	+	–
56	CLAW	t5428	CC522-ST3045	–	–	–	–	–	–	–	+	+	+	+	–	–
57	CLAW	t5428	nd	–	–	–	–	–	–	–	+	+	+	+	–	–
58	CLAW	t14054	CC130-ST700	–	–	–	–	–	–	–	+	+	+	+	–	–
59	CLBW	t5428	CC522-ST522	–	–	+	+	–	–	–	+	+	+	+	+	–
60	CLBW	t5428	nd	*sec, sel*	–	–	–	–	–	–	+	+	+	+	+	–
61	CLBW	t5428	nd	–	–	–	–	–	–	–	+	+	+	+	+	–
62	CLBW	t5428	nd	–	–	–	–	–	–	–	+	+	+	+	–	–
63	CLBW	t5428	nd	–	–	–	–	–	–	–	+	+	+	+	–	–
64	CLBW	t5428	nd	–	–	–	–	–	–	–	+	+	+	+	–	–
65	CLBW	t7630	CC522-ST522	–	–	–	–	–	–	–	+	+	+	+	+	–
66	CLBW	t7630	nd	–	–	–	–	–	–	–	+	+	+	–	–	–
67	CLBW	t14052	CC522-ST522	–	–	–	–	–	–	–	+	+	+	+	–	–
68	CLS^*^	t899	CC398-ST398	–	–	–	–	–	–	–	+	+	+	+	+	–
69	CLS^*^	t899	nd	–	–	–	–	–	–	–	+	+	–	–	–	–
70	CLS^*^	t899	nd	–	–	–	–	–	–	–	+	+	–	–	–	–
71	CLS	t899	nd	–	–	–	–	–	–	–	+	+	–	–	–	–
72	CLS	t189	CC188-ST188	*sea*	–	–	–	+	+	–	+	+	+	+	–	–
73	CLS	t1255	CC398-ST398	–	–	+	+	–	–	–	+	+	+	+	–	–
74	CLS	t13985	CC72-ST72	*seg, sei*	–	–	–	–	–	–	+	–	+	+	–	–
75	CLI	t524	CC71-ST71	–	–	–	–	–	–	–	+	+	+	+	+	–
76	CLI	t524	nd	–	–	–	–	–	–	–	+	+	+	+	–	–
77	CLAL	t002	CC5-ST3059	*seg, sei*	–	–	–	–	–	–	+	+	+	+	-	-
78	CLAX	t998	CC133-ST133	*sec, sel*	+	–	–	–	–	–	+	–	+	+	+	–
79	CLB	t13269	CC8-ST8	*sed, sej*	–	–	–	–	–	–	+	+	+	+	+	–
80	CLBE	t1532	CC130-ST700	*sec, sel*	+	–	–	–	–	–	+	+	+	+	+	–
81	CLBJ^*^	t127	CC1-ST1	*seh*	–	–	–	–	–	–	+	+	+	+	–	–
82	CLBS	t1773	CC130-ST2011	–	–	–	–	–	–	–	+	–	+	–	+	–
83	CLBX	t091	CC7-ST7	–	–	–	–	–	–	–	+	+	+	+	+	–
84	CLBY	t2678	CC133-ST3058	*sec, sel*	+	–	–	–	–	–	+	+	+	+	+	–
85	CLBZ	t1077	CC425-ST3047	–	–	–	–	–	–	–	+	–	+	+	+	–
86	CLO	t1773	CC130-ST3044	–	–	–	–	–	–	–	+	+	+	+	+	–
87	CLW	t1773	CC130-ST3044	–	–	–	–	–	–	–	+	+	+	+	+	–
88	CLND^**^	t005	CC22-ST22	*seg, sei*	–	–	–	+	+	+	–	+	+	+	–	–

Multi Locus Sequence Typing (MLST) was performed on strains representative of each RS-PCR genotypic cluster and spa type combination.

+, gene detected; –, gene not detected;

*, MRSA strains (6);

**, *not determined cluster; nd, not done; ST, sequence type; CC, clonal complex*.

**Figure 1:**
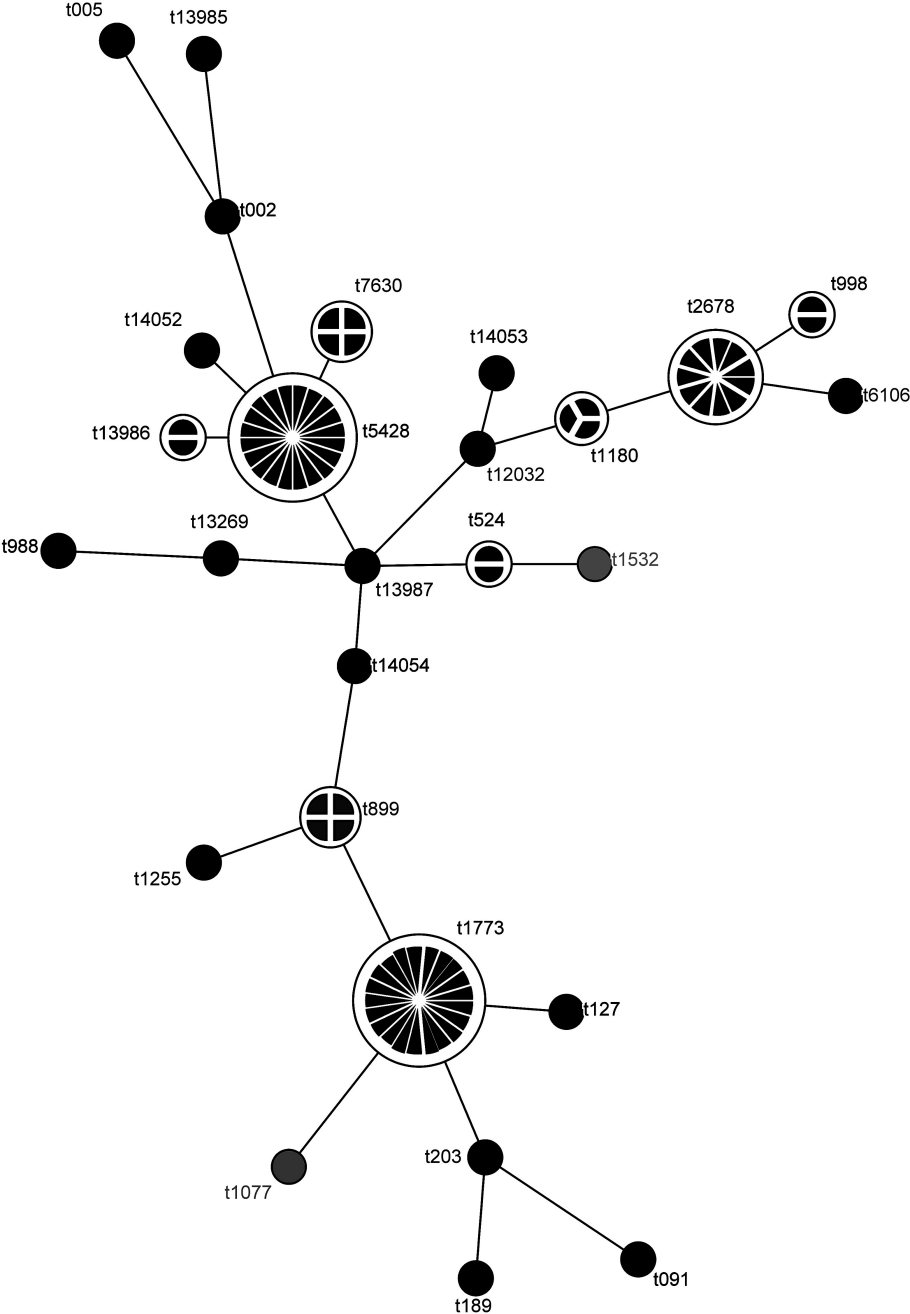
Minimum spanning tree for *Staphylococcus aureus* strains isolated from goat milk. The analysis was based on *spa* typing results and elaborated with BioNumerics 7.6 (Applied Maths, Sint-Martens-Latem, Belgium). The size of the circles and the number of sections reflect the numerosity of each *spa* type.

Using the observed *spa* types, a MST was constructed. MST revealed 3 major *spa* types (t1773, t2678, and t5428) that were linked to a few secondary and tertiary *spa* types. In between there was a set of rarely observed *spa* types, some of them were involved in connecting the 3 main types (Figure 1).

### Virulence Factors

An overview of the prevalence of the most important virulence genes carried by the 88 considered strains is provided in Table 2. Overall, 44.3% (*n* = 39) of all isolates harbored at least one enterotoxin gene. The most frequent was the combination *sec*-*sel*, which was present in 35.2% (*n* = 31) of the isolates, followed by *sed*-*sej* (*n* = 3; 3.4%), *seg*-*sei* (*n* = 3; 3.4%), *sea* (*n* = 1; 1.1%), and *seh* (*n* = 1; 1.1%). The molecular analysis showed that *tst, eta*, and *etb* genes were detected in 31.8, 2.3, and 2.3% of the isolates, respectively. Most of the isolates was positive for genes related to host adhesion and invasion, such as *fmtB* (*n* = 86; 98%), *clfA* (*n* = 85; 96.6%), and *cna* (*n* = 71; 80.6%), whereas few isolates carried genes of the immune evasion cluster (*scn, n* = 2, 2.3%; *sak, n* = 2, 2.3%; *chp, n* =1, 1.1%). Genes encoding *lukE*-*lukD* and *lukM* were observed in 94.3% (*n* = 83) and 60.2% (*n* = 53) of isolates, respectively. None harbored the gene encoding Panton-Valentine leukocidin.

### MLST

Thirty-nine isolates were selected to perform a MLST analysis. The isolates were assigned to 23 different ST, which belonged to 14 different CC. One ST was a singleton (Table 2). The most frequent CC were CC133 (*n* = 10; 25.6%), CC130 (*n* = 9; 23.1%), and CC522 (*n* = 7; 17.9%). CC398 was detected in 5.1% of isolates, whereas each of the remaining CC was assigned to only one isolate.

**Figure 2:**
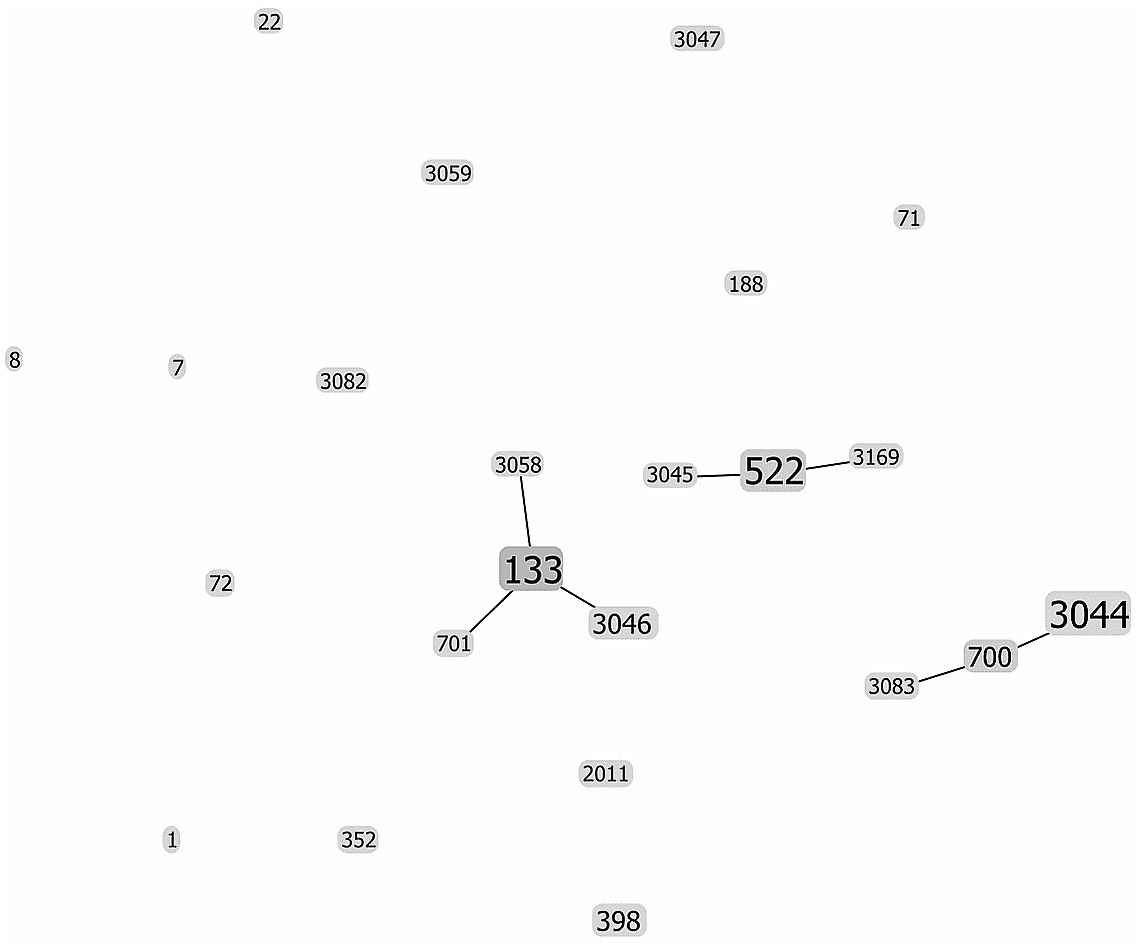
Evolutionary relationship between the different ST of *S. aureus* strains isolated from goat milk. The analysis was performed with goeBURST (Algorithm 3.0), which predicts the founding (ancestral) genotype of each ST. The size of each square reflects the number of strains. The ST located in the periphery of the founder CC are the corresponding single locus variants. The remaining STs are unrelated.

The evolutionary relationships between the different goat *S. aureus* strains are displayed in Figure 2. Three central ST were observed (ST133, ST522, and ST700), corresponding to CC133, CC522, and CC130, respectively. These clones are the founders of additional single locus variants (Figure 2), reflecting a further evolution away from the central clones. All the other ST/clones remain unrelated.

### Statistical Analysis

PSI was estimated by comparing the RS-PCR genotypes of the strains (18 different *S. aureus* CL), isolated from the 65 caprine BTM samples in our study, with the 471 strains (44 different CL) isolated from 398 bovine BTM samples in a recent study (12). The index was 0.311, indicating a very low correlation between the distribution of caprine and bovine *S. aureus* genotypes.

## Discussion

Several studies used RS-PCR to genotype *S. aureus* strains isolated from bovine milk, yet no information on *S. aureus* from goat milk is currently available. For the first time, we genotyped via RS-PCR 295 *S. aureus* isolates, which grouped in 18 genotypic clusters. The major *S. aureus* CL have also been detected in bovine milk, except for CLAW. Other minor CL were here identified and reported for the first time, such as CLW, CLAL, CLBS, and CLBX. Previous studies demonstrated that *S. aureus* CLB, which is the most prevalent among bovine isolates, is highly contagious and associated with high within-herd prevalence of IMI (8, 11, 12). On the contrary, we isolated *S. aureus* CLB in a single BTM from a dairy goat farm located in the province of Sondrio. Furthermore, *S. aureus* CLR and CLS have also been reported to be associated with high within-herd prevalence of IMI (8, 12). *S. aureus* CLR (27.7%) was the most frequently isolated among the analyzed herds. Albeit the lack of details about the sampled farms, we cannot exclude the possibility that some of these farms reared goats and cattle in close proximity, facilitating the spread between the two animal populations.

Most of the isolates were assigned to CC133, CC130, and CC522, so that these lineages may be the major CC among caprine *S. aureus* isolates in Italy as well. Our results are in agreement with previous studies reporting the predominance of these CC in *S. aureus* isolated from milk of small ruminants (4, 15–17).

Typing of *spa* showed a remarkable heterogeneity among *S. aureus* isolates. The most prevalent *spa* types were t1773, t5428, and t2678, corresponding to CC130, CC522, and CC133, respectively, as already reported (16, 17, 30). Comparing RS-PCR genotypes to *spa* types, frequently *Staph. aureus* belonging to CLAW (73%) or CLR (50%), especially to its variants R^III^ and R^VII^, corresponded to t1773. Similarly, *S. aureus* CLAA (75%) or CLBW (67%) corresponded to t5428.

Several studies consider *S. aureus* from goats and sheep to be part of the same population based on CC, since they show pronounced adaptation to small ruminants, but not especially to sheep or goats (17, 30). Nevertheless, different *S. aureus spa* types are reported to be associated with the two species. For example, among CC133 *S. aureus, spa* types t544, t3583, and t7304 were predominantly associated with caprine mastitis, whereas t2678 and t9088 with ovine mastitis (16, 18, 30). However, these *spa* types are not exclusively associated with one host species (30). Actually, CC133/t2678 was one of the most prevalent *spa* types among our strains, whereas we did not find any CC133/t544 or CC133/t3585. Previous studies have described CC133/t544 isolates in sheep as well (16, 18). These contrasting results support the idea that *S. aureus* lineages found among sheep and goats are highly similar (17, 31) and this might be due to the fact that in many countries sheep and goats are farmed together, as previously suggested by (31).

Bar-Gal et al. (15) demonstrated that *S. aureus* isolates from small ruminants differ from the bovine strains in their virulence gene patterns. In fact, cow isolates showed a higher rate of the tested virulence genes than goat and sheep isolates. In our study, 44.3% of all isolates carried at least one enterotoxin gene. All isolates harboring *tst* also carried the genes *sec* and *sel*, and were assigned exclusively to CC130 and CC133, in agreement with previous findings (17, 32). These genes are located on the *S. aureus* pathogenicity island *SaPIov1* (33). Genes coding for toxins usually associated with human diseases, such as *eta* and *etb* (responsible for the staphylococcal scalded-skin syndrome), or *pvl* (leukocidin produced by *S. aureus* that causes leukocyte destruction and tissue necrosis) were rarely or never detected. Moreover, we found high prevalence of leukocidins or genes related to host adhesion and invasion, suggesting their important role as virulence factors for *S. aureus* strains in small ruminants.

The comparison between *S. aureus* strains isolated from goat and bovine milk showed that *S. aureus* from goat milk have wide genetic variability and differ from the bovine strains.

Caprine *S. aureus* is characterized by 3 major *spa* types and a set of rarely observed types, representing a limited genetic variation among caprine strains. Except for t002 and t005, all the observed *spa* are observed frequently, yet are rarely present in the *spa* type database [Ridom SpaServer (see text footnote 2)] implying some uniqueness of the strains analyzed in the present study. Indeed, except for t002 and t005, all remaining *spa* types do not match with those typically observed for human and bovine strains (34, 35). We suspect that t002 and t005 are likely a result of human milk contamination that can occasionally occur during milking and/or equipment cleaning operations. The most prevalent human *spa* types include t032, t003, t002, t008, t011, and t127 (see text footnote 2), whereas those most often observed in bovine milk include t529, t2953, t1403, t524, t024, and t164 (35).

## Conclusion

Our study supports the idea that *S. aureus* from small ruminants constitutes a distinct population in northern Italy, in agreement with previous findings reported worldwide. It also provides for the first time insights to the *S. aureus* RS-PCR genotypes circulating in goat herds in this area, allowing a comparison to bovine strains and adding useful data for epidemiological studies. Further information about *S. aureus* strains isolated from small ruminants is required, as they are a major problem for animal health and may also represent a serious concern for human health, as many dairy products are made from raw milk.

## Data Availability Statement

The raw data supporting the conclusions of this article will be made available by the authors, without undue reservation.

## Interpretative Summary

*Staphylococcus aureus* is the major pathogen responsible for intramammary infections in goats, causing severe economic losses in dairy farms. Furthermore, it can contaminate milk and dairy products, being a source of food poisoning. Since data on the genetic traits and population structure of *S. aureus* from goats are still very limited, our work aimed at determining genetic lineages and virulence traits of *S. aureus* isolated from goat milk in northern Italy. Previous studies used the rapid screening method RS-PCR to genotype *S. aureus* strains from bovine milk, showing a great variety of genotypes differing in their contagiousness. For the first time, we performed RS-PCR on *S. aureus* isolated from goat milk, in addition to more-recognized genotyping methods, such as *spa* typing, MLST, and we investigated their virulence profiles. *S. aureus* isolates showed a wide genetic variability and they differed from bovine strains. Our results support the idea that *S. aureus* from goats may constitute a distinct population, in agreement with previous findings reported worldwide. Our study provides for the first time an overview of the *S. aureus* RS-PCR genotypes circulating in goat herds in northern Italy, adding useful data for further epidemiological studies.

## Author Contributions

ML conceived and planned the experiments. AR, VB, CC, PC, and AG carried out the experiments. AM and FV contributed to the experiments and the interpretation of the results. HG and AR performed the statistical and population study analyses. AG and AR wrote the manuscript in consultation with ML and PC. All authors discussed the results, critically revised, and approved the final manuscript.

## Conflict of Interest

The authors declare that the research was conducted in the absence of any commercial or financial relationships that could be construed as a potential conflict of interest.

## Footnotes

^1^https://mahal.vitech.dev/#/

^2^www.spa.ridom.de

^3^http://saureus.mlst.net.

^4^http://www.mlst.net/eburst/.

^5^https://www.phyloviz.net/goeburst/
