# Supplementary material for: Staphylococcus aureus From Goats Are Genetically Heterogeneous and Distinct to Bovine Ones
Source: Front Vet Sci. 2020 Sep 9;7:628. doi: 10.3389/fvets.2020.00628 (PMC7509144; doi:10.3389/fvets.2020.00628)
Supplement: Supplementary file 1 [file Table_1.DOCX]

Supplementary Table

List of primers and protocols used to investigate the presence of virulence factors that contribute to *Staph. aureus* pathogenicity: enterotoxins (from *sea* to *see*, and from *seg* to *sel*), toxic shock syndrome (*tst*) and exfoliative (*eta*, *etb*) toxins, PVL (*lukS-lukF*), leucocidin M (*lukM*) and leukotoxin ED (*lukE-lukD*), cell-wall associated protein *clfA* (clumping factor A), *cna* (collagen-binding protein), and *fmtB* (cell-wall protein), *scn*, *chp* and *sak* belonging to the immune evasion cluster (IEC).

| **PCR Program ᵃ** | **Targeted gene** | **Name of the primer** | **Amplicon size (bp)** | **Primer sequence (5'-3')** | **References** |
| --- | --- | --- | --- | --- | --- |
| 1 | *sea* | SEA-F1170 | 180 | TAAGGAGGTGGTGCCTATGG | Cremonesi et al., 2005 |
|  |  | SEA-R1349 |  | CATCGAAACCAGCCAAAGTT |  |
|  | *sed* | SED-F578 | 339 | TCAATTCAAAAGAAATGGCTCA | Cremonesi et al., 2005 |
|  |  | SED-R916 |  | TTTTTCCGCGCTGTATTTTT |  |
|  | *seh* | SEH-F260 | 463 | TCACATCATATGCGAAAGCAG | Cremonesi et al., 2005 |
|  |  | SEH-R722 |  | TCGGACAATATTTTTCTGATCTTT |  |
|  | *sej* | SEJ-F349 | 306 | GGTTTTCAATGTTCTGGTGGT | Cremonesi et al., 2005 |
|  |  | SEJ-R654 |  | AACCAACGGTTCTTTTGAGG |  |
| 2 | *sec* | SEC-F97 | 371 | ACCAGACCCTATGCCAGATG | Cremonesi et al., 2005 |
|  |  | SEC-R467 |  | TCCCATTATCAAAGTGGTTTCC |  |
|  | *seg* | SEG-F322 | 432 | CCACCTGTTGAAGGAAGAGG | Cremonesi et al., 2005 |
|  |  | SEG-R753 |  | TGCAGAACCATCAAACTCGT |  |
|  | *sei* | SEI-F71 | 529 | CTCAAGGTGATATTGGTGTAGG | Cremonesi et al., 2005 |
|  |  | SEI-R637 |  | CAGGCAGTCCATCTCCTGTA |  |
|  | *sel* | SEL-F158 | 240 | CACCAGAATCACACCGCTTA | Cremonesi et al., 2005 |
|  |  | SEL-R397 |  | CTGTTTGATGCTTGCCATTG |  |
| 3 | *seb* | SEB-1 | 478 | TCGCATCAAACTGACAAACG | Akineden et al., 2001 |
|  |  | SEB-2 |  | GCAGGTACTCTATAAGTGCC |  |
|  | *see* | SEE-1 | 170 | TAGATAAGGTTAAAACAAGC | Akineden et al., 2001 |
|  |  | SEE-2 |  | TAACTTACCGTGGACCCTTC |  |
| 4 | *tst* | TSST-1 | 350 | ATGGCAGCATCAGCTTGATA | Akineden et al., 2001 |
|  |  | TSST-2 |  | TTTCCAATAACCACCCGTTT |  |
|  | *eta* | ETA-1 | 119 | CTAGTGCATTTGTTATTCAA | Akineden et al., 2001 |
|  |  | ETA-2 |  | TGCATTGACACCATAGTACT |  |
|  | *etb* | ETB-1 | 200 | ACGGCTATATACATTCAATT | Akineden et al., 2001 |
|  |  | ETB-2 |  | TCCATCGATAATATACCTAA |  |
| 5 | *sak* | SAR2039 Fw | 403 | TGAGGTAAGTGCATCAAGTTCA | Sung et al , 2008 |
|  |  | SAR2039 Rv |  | CCTTTGTAATTAAGTTGAATCCAGG |  |
|  | *scn* | SAR2035 Fw | 320 | ATACTTGCGGGAACTTTAGCAA | Sung et al , 2008 |
|  |  | SAR2035 Rv |  | TTTTAGTGCTTCGTCAATTTCG |  |
| 6 | *fmtb* | SAR2248 Fw | 725 | AATGAAGATGCGAATCATGTTG | Sung et al., 2001 |
|  |  | SAR2248 Rv |  | CATCCATTTTTGTTTGCGTAGA |  |
|  | *chp* | SAR2036 Fw | 404 | TTTTTAACGGCAGGAATCAGTA | Sung et al , 2001 |
|  |  | SAR2036 Rv |  | TGCATATTCATTAGTTTTTCCAGG |  |
| 7 | *cna* | cna1 | 192 | AAAGCGTTGCCTAGTGGAGA | Zecconi et al., 2005 |
|  |  | cna2 |  | AGTGCCTTCCCAAACCTTTT |  |
| 8 | *clfa* | ClfA-1 | 1000 | GGCTTCAGTGCTTGTAGG | Akineden et al., 2001 |
|  |  | ClfB-2 |  | TTTTCAGGGTCAATATAAGC |  |
| 9 | *lukE-lukD* | LUKDE-1 | 269 | TGAAAAAGGTTCAAAGTTGATACGAG | Jarraud et al., 2002 |
|  |  | LUKDE-2 |  | TGTATTCGATAGCAAAAGCAGTGCA |  |
| 10 | *lukM* | LUKM-1 | 780 | TGGATGTTACCTATGCAACCTAC | Jarraud et al., 2002 |
|  |  | LUKM-2 |  | GTTCGTTTCCATATAATGAATCACTAC |  |
| 11 | *pvl* | LUKPV1 | 433 | ATCATTAGGTAAATAGTCTGGACATGATCCA | Lina et al., 1999 |
|  |  | LUKPV2 |  | GCATCAASTGTATTGGATAGCAAAAGC |  |

ᵃ PCR Programs:

Both initial denaturation and final extension steps were the same in all reactions: 95°C for 15 minutes, and 72°C for 10 minutes, respectively. Denaturation, primer annealing and extension, and number of cycles varied depending on each reaction:

n. 1, 2, 8: 30 cycles (94°C for 1 minute, 56°C for 1 minute, 72°C for 1 minute);

n. 3: 30 cycles (94°C for 1 minute, 55°C for 2 minutes, 72°C for 1 minute);

n. 4: 35 cycles (94°C for 2 minutes, 55°C for 2 minutes, 72°C for 1 minute);

n. 5, 6: 35 cycles (94°C for 30 seconds, 55°C for 30 seconds, 72°C for 2 minutes);

n. 7: 40 cycles (94°C for 30 seconds, 52°C for 30 seconds, 72°C for 30 seconds);

n. 9, 10: 35 cycles (94°C for 1 minute, 56°C for 1 minute, 72°C for 1 minute);

n. 11: 30 cycles (95°C for 1 minute, 55°C for 1 minute, 72°C for 1 minute).
